# Regulation of Brain-Derived Neurotrophic Factor and Growth Factor Signaling Pathways by Tyrosine Phosphatase Shp2 in the Retina: A Brief Review

**DOI:** 10.3389/fncel.2018.00085

**Published:** 2018-03-27

**Authors:** Mojdeh Abbasi, Vivek Gupta, Nitin Chitranshi, Yuyi You, Yogita Dheer, Mehdi Mirzaei, Stuart L. Graham

**Affiliations:** ^1^Faculty of Medicine and Health Sciences, Macquarie University, Sydney, NSW, Australia; ^2^Save Sight Institute, University of Sydney, Sydney, NSW, Australia; ^3^Australian Proteome Analysis Facility, Macquarie University, Sydney, NSW, Australia; ^4^Department of Molecular Sciences, Macquarie University, Sydney, NSW, Australia

**Keywords:** Shp2 phosphatase, retina, BDNF neurotrophin, growth factors, TrkB receptor

## Abstract

SH2 domain-containing tyrosine phosphatase-2 (PTPN11 or Shp2) is a ubiquitously expressed protein that plays a key regulatory role in cell proliferation, differentiation and growth factor (GF) signaling. This enzyme is well expressed in various retinal neurons and has emerged as an important player in regulating survival signaling networks in the neuronal tissues. The non-receptor phosphatase can translocate to lipid rafts in the membrane and has been implicated to regulate several signaling modules including PI3K/Akt, JAK-STAT and Mitogen Activated Protein Kinase (MAPK) pathways in a wide range of biochemical processes in healthy and diseased states. This review focuses on the roles of Shp2 phosphatase in regulating brain-derived neurotrophic factor (BDNF) neurotrophin signaling pathways and discusses its cross-talk with various GF and downstream signaling pathways in the retina.

## Introduction

SH2 domain-containing tyrosine phosphatase-2 (Shp2) is a 593 amino acid non-transmembrane protein tyrosine phosphatase (PTP) encoded by *PTPN11* gene (He et al., 2014). This phosphatase is ubiquitously expressed and involves the Src homology 2 (SH2) domain facilitating its interactions with phospholipids and phosphoproteins in response to endogenous ligands such as hormones, growth factors (GFs) and cytokines (Dance et al., 2008).

**Figure 1:**
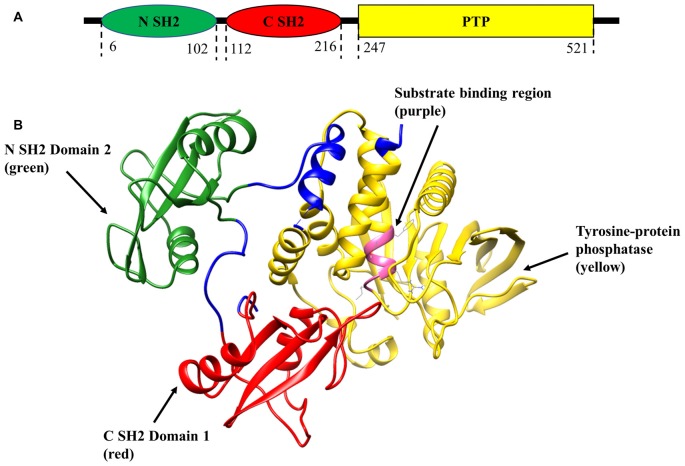
Schematic representation of SH2 domain-containing tyrosine phosphatase-2 (Shp2) structure (PDB:2SHP). **(A)** Two-dimensional structure of tyrosine phosphatase Shp2 comprises of N-terminal Src homology 2 (SH2) domain (green), C-SH2 (red) and protein tyrosine phosphatase (PTP) domain (yellow).** (B)** Ribbon diagram of the crystal structure of full-length Shp2 showing beta sheets and alpha helices. N SH2 domain is shown in green, C SH2 domain in red, PTP domain in yellow and substrate binding region in pink.

Shp2 plays prominent biological roles in regulating several signal transduction cascades affiliated with its function in the early development of vertebrates, cell proliferation, differentiation, transcription regulation and metabolic control (London et al., 2012). Shp2 dysregulation has been stated to be associated with cardiovascular (Lauriol et al., 2015) as well as neurodegenerative disorders of brain and eye (Gupta et al., 2012a; Gómez del Rio et al., 2013). Phosphatase upregulation has been linked to juvenile myelomonocytic, acute myeloid leukemia, and progression of various types of cancers (Mohi and Neel, 2007; Jiang and Zhang, 2008). Dysregulation and inactivation of Shp2 in lower vertebrates leads to severe developmental defects and abnormalities of the central nervous system (CNS), heart and mammary gland (Grossmann et al., 2009). With respect to its structure, Shp2 molecule is comprised of a single catalytic PTP domain, two tandemly arranged SH2 domains in the N-terminal and a carboxy terminal hydrophobic tail (Figure 1A; Neel et al., 2003; Ostman et al., 2006). Both SH2 domains are involved in selectively discerning phosphorylated sites on other molecules and binding to them, thereby mediating Shp2 interactions with different receptors and adaptor proteins (Tartaglia et al., 2001; Li et al., 2016) while the C-terminal tail might promote protein-protein interactions (Neel et al., 2003). Crystal structural studies of Shp2 phosphatase revealed that under normal condition N-SH2 domain exhibits an intramolecular interaction with the PTP active site, thereby auto-inhibiting the Shp2 catalytic activity (Neel et al., 2003; Tartaglia and Gelb, 2005). However, upon Shp2 engagement by tyrosine-phosphorylated proteins, a conformational change in the domain would relieve the auto-inhibitory effects, thereby unlocking the Shp2 phosphatase activity (Figure 1B; Neel et al., 2003; He et al., 2014).

This phosphatase is well expressed in various regions of the brain such as the cerebellum, brain cortex and hippocampus (Rusanescu et al., 2005). Intracellular signaling mediated by Shp2 has found to be crucial in mediating neural cell-fate decisions through nervous system development to ensure that cortical precursor cells generate neuronal cells rather than a glial cell type during brain development, while its neuroprotective actions are reported to be directed against ischemic brain injury (Ke et al., 2007; Cai et al., 2010).

In the retina, Shp2 is well expressed in the ganglion cell layer (GCL) and inner nuclear layer (INL) and its reactivity has been detected in photoreceptors (Kinkl et al., 2002). Shp2 is suggested to be involved in neuronal morphogenesis during early embryonic stages while during postnatal development no further deficits in retinal differentiation were observed in Shp2 mutants indicating a critical role of the protein during early retinal development (Cai et al., 2010). Retinal degenerative changes particularly localized to the inner retina along with optic nerve atrophy in Shp2 ablated rodent models reinforces the important role played by Shp2 in the retina (Cai et al., 2011; Pinzon-Guzman et al., 2015). Shp2 was further demonstrated to mediate Sema4D repulsive signaling to provide axonal guidance in the embryonic chick and mice retinas (Fuchikawa et al., 2009). Numerous *in vitro* and *in vivo* studies have addressed involvement of Shp2 phosphatase and its functional and biochemical effects in various cell signaling pathways in-depth. Here we discuss the modulation of the brain-derived neurotrophic factor (BDNF) as well as multiple GF signaling networks by the Shp2 phosphatase and its implications in the retina.

## Role of SHP2 in BDNF Mediated Survival Signaling

Neurotrophins (NTs) are secreted proteins that regulate neural growth, survival and function by negatively affecting the induction of various cellular apoptotic pathways. Retinal ganglion cells (RGCs) express various neurotrophic factors and are also supported by neurotrophic factors obtained locally from the Muller cells and retrogradely from the brain through axonal flow (Takihara et al., 2011). The neuromodulatory effects of BDNF in particular, play an important role in neuronal regeneration, development, maintaining the health of RGCs and protecting them from apoptosis (Cheng et al., 2002; Nakazawa et al., 2002).

Neurotrophin-regulated signaling cascades have been shown to protect the RGCs and suppress apoptotic pathways (Liu et al., 2002; Gupta et al., 2013). BDNF is a high-affinity ligand of tropomyosin-related kinase B (TrkB) and is shown to be effective in suppressing RGC death caused by axotomy or axonal injury in rodent models (Notaras et al., 2017).

**Figure 2:**
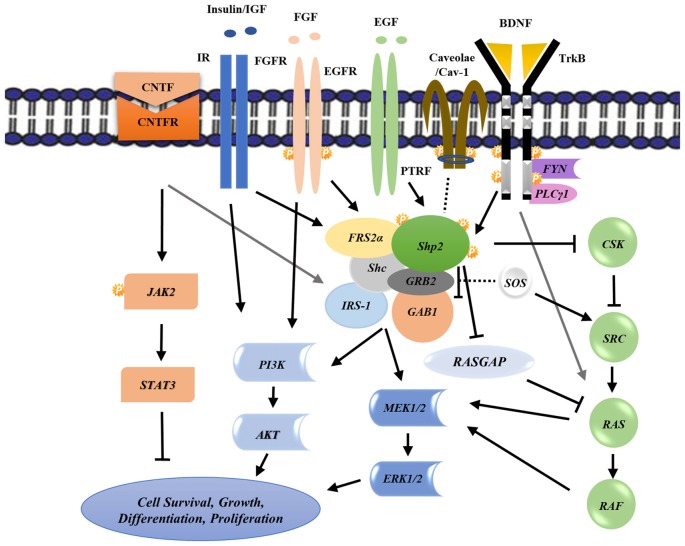
Schematic representation of various biochemical intracellular signaling pathways involving Shp2 and its cross talk with other receptors leading to downstream effects on cell survival, growth, differentiation and proliferation. Arrows and T-bars indicate positive and negative regulations respectively while the dash lines show the possible interactions. See the text for regulation details. P, Phosphorylation; CNTF, Ciliary neurotrophic factor; IR, Insulin receptor; FGF, Fibroblast growth factor; EGF, Epidermal growth factor; TrkB, Tropomyosin-related kinase B; FRS2, FGF receptor substrate 2; GAB1, GRB2-associated binder-1; GRB2, Growth factor receptor-bound protein 2; Shc, Src homology 2 domain containing; CSK, C-terminal Src kinase; GAPs, GTPase-accelerating proteins; ERK, Extracellular signal-regulated kinases; MEK, Mitogen-activated protein kinase kinase; PI3K, phosphoinositide 3-kinase.

BDNF induces Shp2 phosphorylation and its subsequent association with adaptor proteins including Fibroblast growth factor receptor substrate 2 (FRS2; FGF receptor substrate 2; Easton et al., 2006), Src homology 2 domain containing (Shc; Gupta et al., 2013) as well as GRBrb2/SOS for complete Mitogen Activated Protein Kinase (MAPK) activation (Figure 2; Easton et al., 2006; Gupta et al., 2013), suggests a role of this protein in mediating scaffold functions (Chitranshi et al., 2017a). MAPK activation is shown to be neuroprotective in glaucoma conditions (Cai et al., 2011) where neurodegeneration leads to irreversible vision defects (Gupta et al., 2013). A consensus amino acid sequence, NPXY motif, in TrkB sequence is recognized by the phosphotyrosine binding (PTB) domain of Shp2, and is indispensable for Shp2-FRS2 association (Easton et al., 2006). This interaction helps mediate the pathway in a BDNF-dependent manner (Kumamaru et al., 2011). Shp2 may thus potentially function as a transducing protein connecting BDNF and TrkB to MAPK activation and suggests a positive role of Shp2 in regulating MAPK signaling pathway (Figure 2; Easton et al., 2006; Kumamaru et al., 2011).

The phosphatase also plays important roles in the early development of the retina. Cai et al. (2010) demonstrated an essential role of Shp2 phosphatase in retinal cell fate during optic vesicle formation of early embryonic period although its deletion was not found to influence retinal development after initiation of retinal differentiation. Retinas with mutant Shp2 showed features of retinal gliosis, progressive apoptosis of all retinal cell types and other degenerative changes suggesting its important role in Muller glial cells (Cai et al., 2011). Dysfunction of Muller glial cells due to genetic disruption of Shp2 may indirectly affect other retinal neurons such as photoreceptors and RGCs leading to retinal neuronal death and degeneration (Joly et al., 2008; Bringmann et al., 2009). This fact is due to the neuroprotective support that Muller cells provide to the whole retina potentially by producing neuroprotective factors such as BDNF that enhance neuroprotective survival signaling through Extracellular signal-regulated kinases (ERK) and Akt pathway activation (Bringmann et al., 2009; Table 1). Genetic disruption of Shp2 resulted in aberrant ERK phosphorylation in Muller cell bodies within the GCL and INL with extensive retinal degeneration and optic nerve dystrophy. K-ras activation however partially rescued retinal loss suggesting that Shp2 might act in a Ras-MAPK dependent signaling pathway (Cai et al., 2010, 2011; Table 1).

**Table 1:** Involvement of Shp2 phosphatase in three major intracellular signaling pathways (A) JAK-STAT (B) RAS-MAPK (C) PI3K-Akt and their effects in the retina.

	Shp2 associated growth factor signaling in the retina
**A**		
	Indirectly required for Photoreceptor survival	JAK-STAT3 signaling (Cai et al., 2011)
	Physiological pruning of ocular hyaloid vessels during eye development	JAK-STAT1 signaling (Salvucci et al., 2015)
JAK-STAT	Inhibition of photoreceptor differentiation in late embryonic retina	CNTF signaling (Ozawa et al., 2004)
	Rod photoreceptor differentiation	IR signaling (Pinzon-Guzman et al., 2015)
**B**		
	Retinal progenitor cell differentiation	FGF signaling (Cai et al., 2011)
	Muller cells maturation and function	FGF signaling (Cai et al., 2011)
Ras-MAPK	Regulation of TrkB receptor under stress condition	BDNF/TrkB signaling (Gupta et al., 2012b)
	Survival mechanism for normal retina	FGF signaling (Gotoh et al., 2004)
**C**		
PI3K-Akt & Ras-MAPK	Early development of lens and retina	FGF signaling (Li H. et al., 2014)

Functions of this phosphatase in sustained activation of Ras/ ERK signaling effectors and its positive involvement in BDNF/TrkB-promoting survival effects on PC12 cells and also on cultured cerebral and ventral mesencephalic neurons have previously been reported (Neel et al., 2003; Zhang et al., 2004). The positive effects of Shp2 on Ras and ERK/Akt signaling pathways can possibly be mediated through its regulatory effects on negative regulators such as modulating Ras-GTPase activating protein or C-terminal Src Kinase (CSK; Figure 2; Zheng et al., 2003).

Depending upon the partners and downstream signaling pathways, Shp2 phosphatase has also been shown to exert dominant regulatory negative effects (Tartaglia and Gelb, 2005). Rusanescu et al. (2005) suggested that BDNF-induced activation of Ras, Akt and ERK is regulated by increased cross-talk between Shp2 and TrkB receptor. This interaction negatively affects TrkB autophosphorylation and its activation. Accordingly, Shp2 deletion resulted in TrkB activation and enhanced the survival rate of glutamate-exposed neural cells (Rusanescu et al., 2005). The negative effects of Shp2 on TrkB activation have also been identified in the RGCs isolated from the animal retina (Gupta et al., 2012b; also see Table 2). An increased Shp2-TrkB interaction was observed under glaucomatous stress conditions indicating a pathological cross-talk between the two proteins while its inhibition restored TrkB activity under the same condition (Harper et al., 2009; Gupta et al., 2012b). TrkB activation has been shown to play a critical role in RGC survival under various stress conditions and therefore Shp2 activation or its enhanced interactions with TrkB are likely to suppress the neuroprotective pathways leading to RGC loss and optic nerve axonal deterioration (Gupta et al., 2012b). The effects of Shp2–TrkB interaction on axonal regeneration is another potential area for investigation under glaucoma conditions.

**Table 2:** The summary on Shp2 function and its biochemical effects in signaling pathways involved in retina.

Model	Shp2 function	Biochemical effects	Reference
Rat retina	Binds to TrkB receptor	Negatively regulates BDNF/TrkB signaling in RGCs	Gupta et al. (2012b), You et al. (2013)
Mice retina	Neuroprotection; neuronal maturation; development	Plays a critical role in maturation and function of Muller cells which is required for retinal neuron survival; positively regulates Ras-ERK signaling in retinal survival	Cai et al. (2011)
Mice	Optic nerve cup patterning during early embryonic development	Initiation of retinal neurogenesis; controls optic vesicle patterning; mediating Ras-FGF signaling	Cai et al. (2010)
Photoreceptors	Reduces STAT3 phosphorylation	Promotes photoreceptor differentiation	Pinzon-Guzman et al. (2015)
Mice retina	Direct binding to Frs2α	Retinal and lens development through FGF signaling	Li H. et al. (2014)
Retinal neurons	TrkB dephosphorylation	Recruited by MAG-induced PIR-B receptor and regulates PIR-B related pathways in neurons; promotes inhibition of neurite growth	Fujita et al. (2011a)
Rat; retina *ex vivo*	Associates with BIT following photo stimulation	Regulates BIT phosphorylation which is involved in neural transmission in the retina	Hamada et al. (2004)
Mice	Recruited by FGFR following FGF stimulation	Early development of lens and retina through signaling	Gotoh et al. (2004)
Photoreceptors	Associated with transducin-a and a 97-kDa tyrosine-phosphorylated protein in ROS	Plays an essential role in photoreceptor signaling pathways by regulating GF-associated downstream signals	Bell et al. (1999)

*Abbreviations: PIR-B, Paired immunoglobulin-like receptor B; MAG, myelin-associated glycoprotein; BIT, brain immunoglobulin-like molecule; ROS, retinal photoreceptor rod outer segments; GF, Growth factor*.

Shp2-TrkB interaction has been demonstrated to be mediated through the adapter protein caveolin-1 (Cav-1), the prominent structural constituent of caveolae (Figure 2). Experimental glaucoma stress conditions caused Cav-1 protein hyperphosphorylation which resulted in increased binding to Shp2 phosphatase in the RGCs (Gupta et al., 2012b; Chitranshi et al., 2017b). Shp2 was shown to affect Cav-1 cellular functions through binding to phosphorylated Cav-1 under various stress conditions, hindering the complex formation among Cav-1 and CSK thereby positively regulating the Src signaling pathway and ERK phosphorylation (Yun et al., 2011; Jo et al., 2014). Phosphorylation of Shp2 has previously been shown to be dependent on the presence of Cav-1. Cav-1 downregulation using small interfering RNA (siRNA) significantly reduced Shp2 tyrosine phosphorylation (Yun et al., 2011). This interaction is shown to be mediated via N-SH2 binding domain of Shp2 but not the C-terminal PTP motif, that regulates the downstream signaling (Park et al., 2015).

The role of other significant caveolar proteins such as Cavin family members particularly polymerase I and transcript release factor (PTRF) which participate in caveolae formation through its interaction with Cav-1, have not been extensively investigated and their potential cross-talk with Shp2 remains to be explored (Hansen et al., 2013).

Our ongoing studies have indicated that Shp2 overexpression in both neuroblastoma cell line (SHSY5Y cells) and in the RGCs *in vivo* lead to an enhanced endoplasmic stress response induction and diminished TrkB activity (Chitranshi et al., 2017a). Overall, these studies might explain the transient effects of TrkB or BDNF modulation in delaying the death of RGC under glaucomatous or stress conditions. BDNF/TrkB is a potent survival pathway in the visual system (Fu et al., 2009). Therefore, the negative regulation of TrkB through Shp2 phosphatase might explain why BDNF/TrkB activation in RGCs has only transient protective effects *in vivo* (Gupta et al., 2012b).

In cerebellar granule neurons, Shp2 suppressing functions are reported to abolish axonal regeneration by paired immunoglobulin-like receptor PIR-B/Shp mediated TrkB inhibition (Fujita et al., 2011a). Myelin-associated glycoprotein (MAG) stimulation resulted in PIR-B mediated recruitment of Shp2 and Shp1. Shp2/Shp1 downregulation using siRNA was sufficient to reduce the MAG induced TrkB dephosphorylation and subsequent neurite growth inhibitory effects caused by MAG/PIR-B signal transduction. The negative regulation of Shp2 on TrkB was also confirmed in dissociated retinal neurons as well as in the animals subjected to optic nerve injury where Shp2 knockdown was contributed to reduced MAG-induced TrkB dephosphorylation levels in RGCs and significant promotion of optic nerve regeneration (Fujita et al., 2011a,b).

Nerve growth factor (NGF), another member of the neurotrophin family, exerts its survival-promoting effects by stimulating neural development and differentiation through MAPK cascade (Lambiase et al., 2002). The pivotal functions of this neurotrophin in the visual system and retina in particular, is highlighted by the expression of its high-affinity receptor TrkA in RGCs, glial and bipolar cells which express NGF that provide the protective effects on these neurons against various diseases (Wang et al., 2014). NGF therapies have been helpful in protecting the retina and optic nerve in raised intraocular pressure (IOP) models or in neurodegenerative disorders such as Alzheimer’s disease (AD; Lambiase et al., 2009; Roberti et al., 2014) which shares various similarities in ocular manifestations to glaucoma disease (Mirzaei et al., 2017). The survival effects of NGF on retina have been investigated by several researchers, demonstrating that endogenous and exogenous NGF might be helpful in clinical approaches to treat retinal damage and attenuate RGC degeneration caused by glaucoma (Lambiase et al., 2009; Roberti et al., 2014).

It has been previously shown that NGF binding to TrkA receptor results in enhanced Shp2 phosphatase activity which together with FRS2/FRS3 and GRB2 play a critical role in inducing neurite extension (Dixon et al., 2006; Easton et al., 2006) in the cultured cortical neurons and PC12 cells (Goldsmith and Koizumi, 2002). Yet, the role of Shp2/TrkA interactions or Shp2-mediated effects on NGF signaling in the retina have not yet been explored.

### SHP2 in Growth Factor Signaling

Shp2 phosphatase has also been shown to be involved in regulating FGFR (Cai et al., 2011; Li X. et al., 2014), Epidermal growth factor (EGFR), Insulin growth factor (IGF-1), PDGFR (Zhang et al., 2002), thyroid hormone (Liu et al., 2011) and ciliary neurotrophic factor (CNTF; Ohtani et al., 2000; Ozawa et al., 2004) signaling in the neuronal cells. Deletion of the N-SH2 domain in various studies resulted in embryonic lethality and eliminated MAPK activation. Shp2 as a mediator of activated RTK signaling regulates several important cellular signaling pathways (Table 1). The transforming ability of many GF receptors including FGFR, EGFR and ERBB2 appear to be dependent on this phosphatase (Deb et al., 1998; Nakazawa et al., 2002; D’Alessio et al., 2003; Neel et al., 2003; Liu et al., 2011).

FGF family members are implicated in signaling pathways responsible for vertebrate retinal development, differentiation and lens vesicle patterning where Shp2 functions as a vital downstream mediator (Cai et al., 2010; Li H. et al., 2014). Signaling is initiated via FGF stimulation and the direct interaction of FRS2 mediator to the activated FGFR receptor leads to recruitment of other adaptor proteins including GRB2 and Shp2 phosphatase, thereby activating Ras/ERK signaling pathway (Figure 2). However, the overarching hypothesis suggest that FRS2α phosphorylation sites for Shp2 binding, play major role in the ERK activation and the resultant control of eye development (Gotoh, 2008; Kim et al., 2015; Table 1). Accordingly the FRS2α mutant lacking these binding sites depicted failure in lens and retinal developmental (Li H. et al., 2014).

This phosphatase, consequently, has proven roles in providing neuroprotection, maintaining Muller cell function, directing retinal neuronal fate during early retinal and lens development and regulating intrinsic retinal survival mechanisms while its ablation has been shown to severely disrupt the retinal cell maturation leading to extensive retinal cell death and degeneration (Gotoh et al., 2004; Cai et al., 2010; Li H. et al., 2014). The Akt intracellular signaling process is apparently not involved in FGF-induced developmental processing and it was not able to compensate for the retinal degenerative phenotype linked to Shp2-ablation (Cai et al., 2011).

Shp2 was shown to negatively regulate phosphoinositide 3-kinase (PI3K) signaling in human glial cells in response to EGF treatment. Zhang et al. (2002) demonstrated that Shp2 is involved in regulating duration or strength of PI3K activation and GAB1-mediated PI3K signaling could be activated in fibroblasts expressing mutant Shp2 (Zhang et al., 2002; Mattoon et al., 2004). In contrast, Cai et al. (2011) indicated that although both Shp2 and PI3K are involved in normal retinal protection, these two proteins operated separately showing little cross-talk and enhancing the PI3K signaling did not compensate Shp2 deficits in Shp2 mutant mice retinas. Conversely, PI3K activation reduced upon Shp2 ablation following other GFs stimulation including PDGF and IGF-1, suggesting differential effects of Shp2 in response to various GFs (Zhang et al., 2002). IGF-1 is expressed in retinal pigment epithelium and was shown to play vital roles in the differentiation of cultured retinal neuroepithelial cells in the presence of laminin-1 while its absence or antibody-mediated blocking seriously affected retinal neuronal differentiation (Frade et al., 1999).

The EGF-inhibitory impact of Shp2 was also investigated in a glioma cell line (SNB19). Despite lack of proliferation ability of SNB19 in response to EGF stimulation, it was found that interfering Shp2 mutant could reverse the cell’s ability to proliferate following EGF induction (Reeves et al., 1995). Using mutants depicting various levels of EGFR activity has revealed differential effects of this receptor during retinal development (Oishi et al., 2006). However, in-depth mechanisms of Shp2 dependant EGFR activity in retinal cell development remain to be explored.

Shp2 has also been suggested to negatively regulate Janus kinase-signal transducer and activator of transcription (JAK-STAT) signaling cascade which has major functions in cellular processes such as proliferation, differentiation and apoptosis (Kisseleva et al., 2002). Hee et al. (2006) demonstrated that in the brain microglia, Shp2 is involved in transient stimulation of JAK-STAT signaling following ganglioside induction. This occurs through lipid raft mediated Shp2 phosphorylation and its subsequent association with JAK kinase leading to negative regulation of signaling (Hee et al., 2006). In retinal neurons (rod photoreceptors), Shp2/Shp1 phosphatase is recruited through IGF-1 induced pathway, reduces the level of phosphorylated STAT3 and thereby promotes photoreceptor differentiation (Pinzon-Guzman et al., 2015; Table 2). Interestingly, a novel pathway was recently identified by Salvucci et al. (2015) that highlighted the negative regulation of STAT1 by Shp2 phosphatase (Salvucci et al., 2015). EphrinB2, a critical regulator of retinal vasculature pruning and vessel survival (Salvucci and Tosato, 2012), was demonstrated to be involved in recruitment of Shp2 phosphatase providing physiological pruning of hyaloid vessels during eye development (Salvucci et al., 2015).

Nevertheless, Shp2 ablation, in photoreceptors was shown to stimulate STAT3 activation which might either suggest the regulatory role of phosphatase in photoreceptors survival pathway or be an injury dependant response protecting the retina from further profound injury caused by ERK downregulation. This signaling, although required for cell differentiation in the postnatal retina, is dispensable for retinal hemeostasis in normal physiological conditions (Cai et al., 2011).

JAK2 and STAT3 effectors have been mainly elucidated in retinal layers, GCL and INL suggesting they mediate neuroprotective activity in ganglion and Muller cells through CNTF stimulation (Peterson et al., 2000). In the late embryonic period and in the postnatal stage, STAT3 is activated through CNTF-mediated gp130 receptor and entirely inhibits differentiation of rod photoreceptors (Table 1, Ozawa et al., 2004; Pinzon-Guzman et al., 2015). CNTF also functions through Shp2-mediated Ras/MAPK downstream pathway involved in cell growth and survival (Hirano et al., 1997; Ohtani et al., 2000). However, activation of STAT3 downstream effector but not Shp2 mediated signaling is required during post-natal retinal development (Ozawa et al., 2004). In addition, phosphatase has a proven role in oligodendrocyte maturation and differentiation (Liu et al., 2011). Shp1 genetic ablation is associated with negative modulation of myelination (Massa et al., 2004) while Shp2 plays a pivotal role in thyroid hormone (T3) dependant oligodendrocyte precursor cells (OPC) maturation in CNS which is regulated through Akt and ERK1/2 pathways (Liu et al., 2011). T3 is predominantly involved in optic nerve OPC differentiation and impacts RGC survival (Baas et al., 2002) which might be attributed to the potential regulatory role of Shp2 on the oligodendrocytes within the optic nerve.

Different studies suggest that Shp2 activity is required for activation of insulin receptor (IR) downstream signaling including Ras, Raf, MEK and MAPK cascade. Insulin receptor substrate 1 (IRS-1), one of the major IR substrate is a multisite docking protein which interacts with SH2 domain-containing proteins such as Shp2. Milarski and Saltiel (1994) showed that IRS-1 and Shc dependent phosphorylation of IR was markedly attenuated following Shp2 mutation in fibroblasts, confirming the crucial role of Shp2 phosphatase in regulating IR activity (Milarski and Saltiel, 1994). IR signaling is vital in light dependant PI3K/Akt cascade which provides neuroprotection to the photoreceptors and rescues them from apoptosis while its deletion leads to stress-mediated degeneration of photoreceptors (Rajala et al., 2013). The association of this receptor with Shp2 in neuronal cells in retina has not been rigorously explored.

### SHP2 Involvement in Retinal Pathological Changes

Shp2 involvement in regulating retinal survival signaling pathways links dysregulation of this phosphatase to various physiological and pathological conditions. Under glaucomatous stress Shp2 leads to preferential RGC degeneration by inhibiting BDNF/TrkB downstream signaling through dephosphorylation and deactivation of TrkB (Gupta et al., 2012b, 2013; You et al., 2013). The potential effects of glaucoma extend well beyond the retina into the optic nerve and higher visual centers in the brain through transneuronal changes (You et al., 2013; Gupta et al., 2016). Loss of BDNF/TrkB signaling is reported to be strongly associated with other neurodegenerative disease including AD, Huntington’s, Parkinson’s (Yin et al., 2008; Baydyuk et al., 2011; Gupta et al., 2013) all of which display characteristics of retinal damage and dysfunction (Muqit and Feany, 2002; Bodis-Wollner, 2009; Gupta et al., 2016). Further investigations are required to explore the potential involvement of Shp2 mutations or associated polymorphisms in various retinal indices in both health and disease conditions. Indeed, many patients with Noonan Syndrome, of whom 50% typically harbor *PTPN11* gene mutation (Tartaglia et al., 2002; Zenker et al., 2004), were identified with ocular abnormalities including fundal changes (Marin et al., 2012), optic disk excavation, enhanced cup to disk ratio and myopia, symptoms which are associated with higher risk of glaucoma and retinal degeneration (Whitmore, 1992; Marin et al., 2012; Lee and Sakhalkar, 2014). Applying genetic ablation of *PTPN11* gene in animal studies resulted in extensive retinal degeneration, cell death and optic neuropathy during various developmental stages highlighting its regulatory function in retinal neuroprotection and progenitor retinal cell fate (Cai et al., 2011; Puri and Walker, 2013). Furthermore, Shp2 plays key roles in regulating Akt/mTOR driven myelination that might reflect its role in multiple sclerosis (MS; Ahrendsen and MacKlin, 2013). Many MS patients suffer from visual loss, optic neuritis and RGC degeneration, however any association between the role of Shp2 in myelination and its possible impact on optic nerve has not been investigated (Gundogan et al., 2011; London et al., 2012). Additional approaches including generating knockout or conditional/ inducible gene ablation in different layers of the retina would shed light on the cell specific molecular mechanism of the phosphatase in the retina.

### Concluding Remarks and Emerging Concepts

Shp2 plays an emerging and important role in the retinal development and its preservation. Preliminary *in vivo* and *in vitro* studies implied that the major effect of the phosphatase is mediated through its regulatory effects on various GFs and their downstream effectors which activates multiple signaling pathways. In this review we outlined and analyzed the existing evidence regarding Shp2 involvement in BDNF and other GF-dependant signaling networks with a specific focus on the retinal neuronal cells.

Although significant breakthroughs in the functional characterization of Shp2 have provided more knowledge of the physiological importance of the phosphatase, other areas of future study including manipulating Shp2 expression in retinal and other neuronal cell lineage or development of specific phosphatase inhibitors will further define the mechanisms through which Shp2 positively or negatively mediates various retinal signaling pathways. Another fascinating aspect would be considering Shp2 as a potential molecular target to modulate other neuronal signaling pathways thereby serving as mechanism-based therapy. The involvement of Shp2 in broad range of biochemical actions may make it a challenge to develop any specific therapeutic strategy to isolate a particular neuroprotective role. However, prospective investigations probing Shp2 protein expression, post-translational modifications, sub-cellular localization and interactome changes will unravel the role of this protein in various neurodegenerative disease and retinal disorders.

## Author Contributions

The review was conceptualized, written and edited by each of the authors. Supervisor: SLG.

## Conflict of Interest Statement

The authors declare that the research was conducted in the absence of any commercial or financial relationships that could be construed as a potential conflict of interest.

## Abbreviations

BDNF: Brain-derived neurotrophic factor
EGFR: Epidermal Growth Factor Receptor
FRS2: Fibroblast growth factor receptor substrate 2
GCL: Ganglion cell layer
MAPK: Mitogen Activated Protein Kinase
NGF: Nerve growth factor
RGCs: Retinal ganglion cells
Trk: Tropomyosin Related Kinase
Shp2: SH2 domain-containing tyrosine phosphatase-2
Shc: Src homology 2 domain containing.
